# Management of chlamydia and gonorrhoea infections diagnosed in primary care using a centralised nurse-led telephone-based service: mixed methods evaluation

**DOI:** 10.1186/s12875-020-01329-0

**Published:** 2020-12-10

**Authors:** Jeremy Horwood, Emer Brangan, Petra Manley, Paddy Horner, Peter Muir, Paul North, John Macleod

**Affiliations:** 1 Centre for Academic Primary Care, Population Health Sciences, Bristol Medical School, University of Bristol, 39 Whatley Road, Bristol, BS8 2PS UK; 2 National Institute for Health Research, Applied Research Collaboration West (NIHR ARC West) at University Hospitals Bristol and Weston NHS Foundation Trust, Bristol, UK; 3 NIHR Health Protection Research Unit (HPRU) in in Behavioural Science and Evaluation, University of Bristol, Bristol, UK; 4 Department of Nursing and Midwifery, University of the West of England, Bristol, UK; 5 Field Service, National Infection Service, Public Health England, Bristol, UK; 6 UNITY Sexual Health, University Hospitals Bristol and Weston NHS Foundation Trust, Bristol, UK; 7 Public Health England South West Regional Laboratory, Bristol, UK

**Keywords:** Sexual health, Chlamydia, Gonorrhoea, Partner notification, Qualitative research, General practice

## Abstract

**Background:**

Up to 18% of genital Chlamydia infections and 9% of Gonorrhoea infections in England are diagnosed in Primary Care. Evidence suggests that a substantial proportion of these cases are not managed appropriately in line with national guidelines. With the increase in sexually transmitted infections and the emergence of antimicrobial resistance, their timely and appropriate treatment is a priority. We investigated feasibility and acceptability of extending the National Chlamydia Screening Programme’s centralised, nurse-led, telephone management (NLTM) as an option for management of all cases of chlamydia and gonorrhoea diagnosed in Primary Care.

**Methods:**

Randomised feasibility trial in 11 practices in Bristol with nested qualitative study. In intervention practices patients and health care providers (HCPs) had the option of choosing NLTM or usual care for all patients tested for Chlamydia and Gonorrhoea. In control practices patients received usual care.

**Results:**

One thousand one hundred fifty-four Chlamydia/gonorrhoea tests took place during the 6-month study, with a chlamydia positivity rate of 2.6% and gonorrhoea positivity rate of 0.8%. The NLTM managed 335 patients. Interviews were conducted with sixteen HCPs (11 GPs, 5 nurses) and 12 patients (8 female). HCPs were positive about the NLTM, welcomed the partner notification service, though requested more timely feedback on the management of their patients. Explaining the NLTM to patients didn’t negatively impact on consultations. Patients found the NLTM acceptable, more convenient and provided greater anonymity than usual care. Patients appreciated getting a text message regarding a negative result and valued talking to a sexual health specialist about positive results.

**Conclusion:**

Extension of this established NLTM intervention to a greater proportion of patients was both feasible and acceptable to both patients and HCP, could provide a better service for patients, whilst decreasing primacy care workload. The study provides evidence to support the wider implementation of this NLTM approach to managing chlamydia and gonorrhoea diagnosed in primary care.

## Background

*Chlamydia trachomatis* and *Neisseria gonorrhoeae* are the two most commonly diagnosed bacterial sexually transmitted infections (STIs) in England, with 218,095 and 56,259 diagnoses reported in 2018, a 6 and 26% increase since 2017 [1]. These infections are often diagnosed and treated late, meaning individuals have longer to pass the infection to others and are more at risk of developing long-term consequences. Chlamydia, which is commonly asymptomatic, causes a substantial burden of disease, in women particularly, including chronic pelvic pain, ectopic pregnancy and infertility [2–4]. Gonorrhoea treatment is threatened by the emergence of antimicrobial resistance, which is now a global public health priority for gonorrhoea [5–7], with three cases of extensively drug resistant *Neisseria gonorrhoeae* identified in England in 2018 [8] and may become a future issue for chlamydia [9]. National guidelines recommend appropriate management includes timely testing and treatment, with the right antibiotic, of both the patient and partner(s) with a test of cure if gonorrhoea is detected and a repeat test at 3–6 months in persons under 25 yrs. treated for chlamydia [10, 11]. Referral of confirmed gonorrhoea cases to a genitourinary medicine (GUM) clinic, due to the complexities of management, is also strongly recommended [11, 12]. These recommendations are essential to prevent reinfection, onward transmission, treatment failure, morbid sequelae, minimise the pool of infection in the population and avert further emergence of anti-microbial resistance [10, 11].

Budget cuts for sexual health services resulting in fewer GUM clinics, means patients are not always able to easily access specialist care [13]. The need to increase STI testing outside of GUM clinics and integrate care across providers has been emphasised [1, 14]. An increasing proportion of STI management is now provided by primary care [15]. However, primary care is not organised or resourced to easily deliver appropriate management of STIs. Partner notification is challenging when partners are patients of other practices and a substantial proportion of STIs diagnosed in primary care may not be managed in line with national guidance [5, 16, 17]. Research suggests only 5–11% of patients treated for gonorrhoea in primary care receive the recommended treatment and most cases were prescribed antibiotics no longer recommended [5]. Antimicrobial resistance has required two revisions to the UK national gonorrhoea treatment guideline in the past 10 years [11, 18] and GPs may not be aware of these revisions due to the infrequency of cases seen. There is also emerging evidence of social inequalities in chlamydia infection, alongside more established evidence of inequalities in gonorrhoea infection [19, 20]. Thus more cases of chlamydia and gonorrhoea are likely to be diagnosed in disadvantaged communities where primary care services are already facing multiple demands [21].

The National Chlamydia screening programme (NCSP) in England, introduced in 2003 offers women and men, aged 15–24 years, opportunistic testing to diagnose and control chlamydia infection [22], managed locally by a centralised, telephone-based, nurse-led, STI management service [22]. A key aim of the NCSP is supporting testing within primary care [23], however this management service had been under-utilised by primary care [24]. We piloted extending the NCSP nurse-led telephone-based service as an option for management of all chlamydia/gonorrhoea primary care tests. We evaluated the feasibility and acceptability to primary care practitioners and patients of using this pathway.

## Methods

### Design

**Fig. 1:**
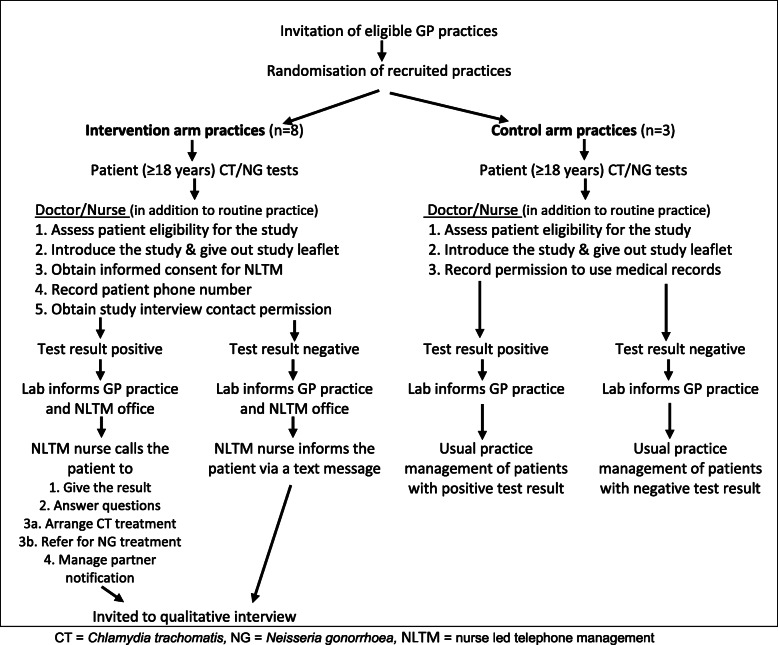
Study design

Primary care practices in Bristol and North Somerset that request laboratory tests through the CliniSys Integrated Clinical Environment (ICE) online system, were invited to participate in the six-month randomised feasibility trial, with cluster randomisation of primary care practices to either intervention (nurse led telephone management - NLTM) or control (usual care) arm (Fig. 1). Practices were randomised by an independent statistician on an 8:3 ratio (intervention: control) to investigate feasibility and acceptability of the intervention. Local data suggested that a sample size of 10–15 practices would be required in order to generate sufficient positive tests to assess feasibility.

Inclusion criteria were patients aged 18 years or over undergoing testing for chlamydia or gonorrhoea at study practices, and able to provide informed consent. There were no additional exclusion criteria, however health care professionals (HCPs) could exclude patients from NLTM where they felt this was inappropriate for clinical or other reasons.

HCPs requested chlamydia and gonorrhoea tests through the ICE online system. We adapted the ICE system so intervention practices could record patient’s consent for NLTM, anonymous data sharing and willingness to be contacted about a qualitative interview. In control practices, the ICE system allowed HCP to record patients’ consent to use anonymised medical records. The testing laboratory notified requesting practices of test results. In the intervention arm, when management by NLTM was chosen, the laboratory also securely emailed patient details to the NLTM office. Patients with negative results received a text from the NLTM and positive patients received a telephone call from a specialist nurse. Any questions were answered, and appropriate treatment via a nominated pharmacy or sexual health clinic arranged along with partner treatment using either the patient or provider led model. Patients with positive gonorrhoea tests were referred to a GUM clinic. The specialist nurse made three attempts to contact patients and extra attempt(s) resulting from clinical assessment of individual cases were also made. Where the nurse was not able to contact the patient, the primary care practice was informed. Data were collected from routine patient record electronic systems.

### Qualitative evaluation

Qualitative semi-structured interviews were conducted with intervention patients and HCPs, to examine views and experiences of NLTM and any barriers to its uptake. Participants were purposively sampled regarding primary care practice, age, gender, test result (patients); and professional role (HCPs). Sample size was driven by the concept of ‘information power’ [25], with continuous assessment of information within our sample with regard to meeting study objective.

A social scientist (EB) invited patients to participate in interviews by phone and HCPs by email. Patient interviews were conducted by phone, and HCPs interviewed face-to-face or by phone. With informed consent (written, or audio for telephone interviews), interviews were audio-recorded, transcribed verbatim, anonymised, imported into NVivo 10 (QSR International) and analysed thematically [26]. A subset of transcripts were independently analysed by EB and JH to contribute to the refinement of codes and maximise rigour. Codes were built into broader categories and themes discussed by the multidisciplinary research team to ensure credibility tests.

## Results

### Quantitative evaluation

11/27 (41%) of practices invited agreed to take part; eight randomised to receive the intervention and three to the control. In control practices 228 of349 tests were submitted amongst recruited patients, mean age 32 years, 121 (34.7%) of patient tests were excluded from the study: 62 patient ineligible, 5 assigned to the incorrect study arm and 54 declined permission for use of anonymised medical records. In intervention practices 436 of805 tests were submitted amongst recruited patients, mean age 34.1 years, 369 (45.8%) patient tests were excluded from the study: 77 clinically inappropriate, 39 patient ineligible, 238 not consented to receive NLTM and 15 were assigned to the incorrect study arm. Interviews with HCPs indicated that few patients declined consent to receive NLTM and it was more usual for the consent process not to be completed due to time pressure during consultations (see ‘Discussing nurse led telephone management’ section).

**Fig. 2:**
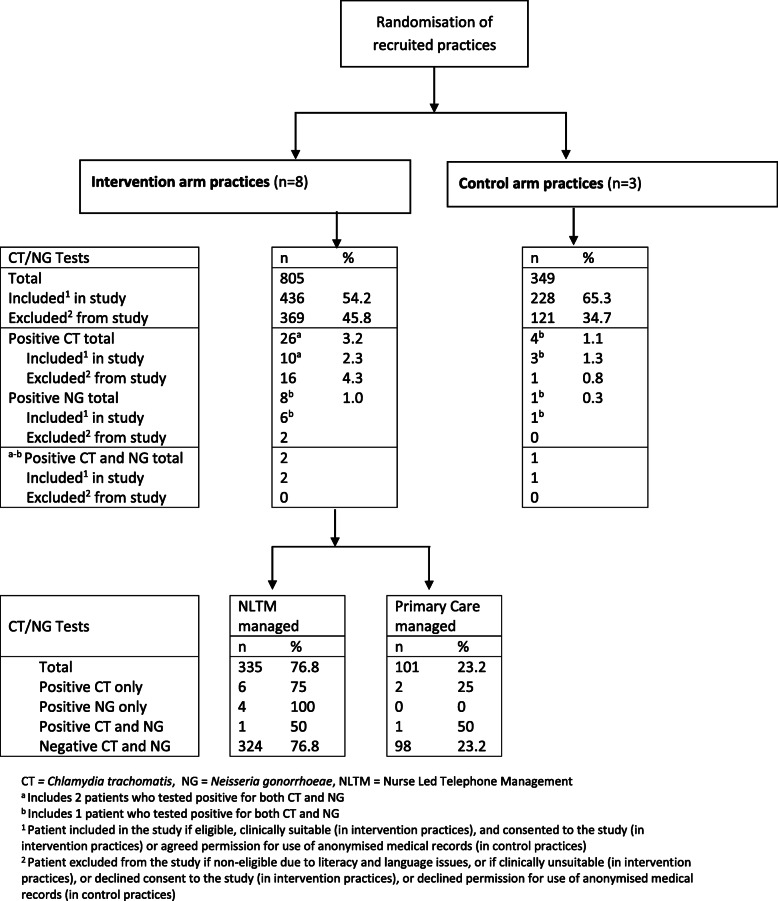
CONSORT Diagram

During the study period (April – September 2015), 1154 Chlamydia/gonorrhoea tests took place, with 805 (70%) in the intervention and 349 (30%) in the control arm (Fig. 2). Thirty tests were positive for chlamydia (positivity rate of 2.6%), and 9 for gonorrhoea (positivity rate of 0.8%). Three patients were positive for both chlamydia and gonorrhoea. The chlamydia positivity rate was slightly lower (2.0, 95% CI: 1.1–3.3) in patients included in the study compared to those excluded (3.5, 95% CI: 2.2–5.5). Approximately 23% (101/436) of patients who consented to receive NLTM were managed via primary care (Fig. 2), qualitative interviews identified that this was due to delays in data transfer to NLTM. This initial issue was resolved by the laboratory automating sending relevant patient details to the NLTM for action. In total the NLTM managed 335 patients, of which 11 tested positive.

### Qualitative evaluation

Sixteen HCPs (11 GPs, 5 nurses) and 12 patients (8 females; ages 22–50/average 32 years; 3 positive test results; ethnicity = 10 White British/1 White other/1 unknown) were interviewed (Table 1). Eleven patients consented to NLTM and one patient was recorded as having declined NLTM but agreed be interviewed. Patients were interviewed between 13 and 39 days (mean = 22 days) after the consultation when their test was taken. Contact was attempted with a further 19 patients who either then declined the interview or were not contactable by telephone despite repeated attempts. Two key themes were developed from the analysis: ‘Views and experiences of the primary care consultation’, and ‘Communicating and managing test results’. Findings are illustrated using anonymised verbatim quotes.

**Table 1:** Interview practice and participant characteristics

Practice	Practice details *(rounded practice population, IMD Xth most deprived decile)*	Average monthly Chlamydia tests^**a**^ *(GP; NCSP)*	HCP participants *(Identity code)*	Patient participants *(Identity code, sex, age, test results)*
**A**	Population 6900 IMD 3rd decile	20 (18; 2)	GP 1 GP 2	Patient 1, female, 50, negative
**B**	Population 10,300 IMD 2nd decile	36 (34; 2)	GP 3 GP 4	Patient 2, male, 22, Positive CT Patient 3, male, 22, negative
**C**	Population 9000 IMD 6th decile	32 (29; 3)	GP 5 Nurse 6	Patient 4, female, 26, negative
**D**	Population 16,000 IMD 6th decile	57 (56; 1)	GP 7 Nurse 8	Patient 5, female, 35, negative
**E**	Population 6000 IMD 1st decile	21 (19; 2)	GP 9 Nurse 10	Patient 6, female, 37, positive CT
**F**	Population 6300 IMD 1st decile	18 (17; 1)	GP 11 GP 12	Patient 7, female, 29, positive NG Patient 8, female, 48, negative Patient 9, male, 25, negative
**G**	Population 7500 IMD 1st decile	39 (37; 2)	Nurse 13 Nurse 14 GP 15	Patient 10, f, 36, negative
**H**	Population 7500 IMD 6th decile	14 (11; 3)	GP 16	Patient 11, m, 24, negative Patient 12, f, 35, negative

*IMD* Index of Multiple Deprivation, 1st decile represents the most deprived [27], *CT* Chlamydia trachomatis, *NG* Neisseria gonorrhoea. ^a^Average based on tests submitted April–September 2014

### Views and experiences of the primary care consultation

#### Consulting primary care for an STI

Patients valued consulting primary care for STIs. Some female patients referred to stigma associated with being seen using sexual health services as motivation for preferring primary care practice.

“*… it’s a bit of a stigma because people know what that specific [GUM] clinic is … and it’s one of those things where, well, what if someone sees me that knows me? They might, you know, and make assumptions”* (Patient 5).

Some patients who tested positive had consulted primary care previously regarding their symptoms before being offered an STI test. Patients thought that opportunities had been missed to diagnose and treat them earlier:

*“I was hoping to have treatment. ‘Cos the first two doctors I’d seen before then, they just kept passing it off as thrush.”* (Patient 7).

#### Discussing nurse led telephone management

Despite the online consent being completed, a minority of patients had no recollection of NLTM being discussed and or had not understood what it involved. Several patients mentioned that the consultation had been in the context of stressful life events, which may have affected their recollection:

*“I can’t remember at all … I was distraught … at the time when I spoke to my doctor.”* (Patient 8)

HCPs mentioned having excluded a patient because their first language was not English, because of patients’ learning difficulties and because the HCP wanted to see the patient for follow up regarding other issues. Another GP decided not to discuss the intervention with some patients she saw as being “*ridiculously anxious already*” (GP 15). The commonest reason intervention practice HCPs gave for not consenting patients related to time pressure/frustration with ICE system online consent procedures, which also contributed to some patients being assigned to an incorrect study arm. If an HCP felt too busy to recruit, they chose the earliest possible exit option on the ICE system (e.g. ineligible or decline consent). The ICE system was subsequently simplified following feedback.

*“Initially … . because it [ICE] was so clunky, and if you were in a rush, it was quicker to press, “No”* (GP 7)

Most HCP did not find explaining the NLTM to patients negatively impacted on consultations. HCPs reported that the vast majority of their patients were happy with NLTM and did not raise any questions or concerns.

*“I usually say, ‘The way positive results are going to be managed is that somebody, with your consent, will contact you from the screening service that already deals with the screening programme, so they’re well used to doing it.’ And most people say, ‘Oh yeah, that’s fine’.”* (GP 5)

One GP did think explaining NLTM, along with the need to check the phone number, would be more time consuming *“as opposed to, ‘Have your result, if it’s positive come back and see me and we’ll sort it out’”* (GP 3). However, they thought that some of this time might be recouped in the longer term via NLTM managing results. Another GP was concerned that, if the STI test was part of a broader diagnostic process, discussing NLTM could put unwanted emphasis on the possibility of a STI:

*“If someone has just come in with a vaginal discharge and they’ve got a regular partner, and you’re saying, ‘I’m going to do some swabs for infection that includes sexually transmitted infection but also includes non-sexually transmitted infection’, to then have to really focus on, you know, ‘A nurse is going to ring you if you’ve got chlamydia’, you’re sort of - it escalates the anxiety about what’s going on. And particularly patients with pelvic pain and stuff that often are very anxious anyway. “(*GP 15)

### Communicating and managing test results

#### Health care professional uncertainties about intervention procedures

Most HCP mentioned uncertainties regarding NLTM procedures, including when the NLTM would contact patients, and when, if at all, the HCP would be informed about what care had been provided, or be notified if the NLTM had not been able to contact the patient. A minority of HCP did not know that patients with negative results were informed by text, and one GP had told patients that they still needed to call the practice. HCPs acknowledged that if NLTM was usual care, rather than a new initiative, they would be likely to have be more confident that the patient was being appropriately managed.

*“if something’s new you’re just not sure how failsafe it is, so I just told people, ‘You must phone us back anyway. But, you know, basically you should receive a phone call from, you know, the specialist hub.’”* (GP 16)

HCP retained a strong sense of personal responsibility for the patients whose tests they had initiated:

*“As the person taking the test, you kind of feel obligated, by the way we’re trained, to follow it up. So, I think that’s what I would do … I guess I might, after the first few positives, I might just become more confident and leave it.”* (GP 4)

A minority of HCP reported that they had checked that a patient had received results and infections were being managed, and others said that they would have done this if a positive result had come back:

*“Well initially maybe it was me who wasn’t aware of how things worked. I mean I think there was one positive result which - I wasn’t sure whether it was being acted upon or not. So, I kind of just did a prescription and, you know, asked the receptionist to find out if he has had a phone call. Because it was a positive result, I just wanted to act upon it … the receptionist got back to me and said that, ‘No, he has already had a [NLTM] phone call.’”* (GP 1)

It was important to HCPs that there was a mechanism to ensure that they were kept fully informed in a timely manner regarding the care their patients were receiving:

*“I didn’t want to just leave it, in case* [NLTM] *didn’t get hold of her. … I think it would be nice to have confirmation that.’”* (Nurse 14)

#### Receiving results

Patients appreciated getting a text message regarding a negative result and found this preferable to a ‘no news is good news’ approach, or to having to try and contact their practice to obtain results.

*“I thought it was good because you get it straight away then, isn’t it?…Trying to get through to a GP surgery is a bit of a nightmare nowadays … it’s good, just have it – peace of mind as well, everything was alright.”* (Patient 1)

Patients in general considered having a telephone consultation regarding positive test results/treatment acceptable, and significantly more convenient and timelier than a face-to-face appointment. Some patients said that they would feel more comfortable having a results conversation by telephone as *“In person it’s quite embarrassing”* (Patient 3). Patient 4 thought that dealing with a positive result would be easier if she was in her “*own environment*”, rather than having to “*hold it all together*” in front of the GP.

#### Appeal of a ‘specialist’ nurse led service

Being contacted by ‘specialist’ nurses was considered attractive by many patients, for a range of reasons. Some patients expected usual care to involve being contacted by primary care reception staff - when checking results or making a follow-up appointment. In one case this increased the patient’s concerns regarding confidentiality:

“…*these receptionists live in the area. And I’m not saying they’ll go and blab things out, but I don’t want them to know my business. So, if it was dealt with by someone who was specialised in that and then you don’t even have to involve the doctor, that’s way better.”* (Patient 8)

Another patient had concerns about the level of expertise needed to communicate results:

*“I always have a bit of doubt when I ring my GP surgery to get results from tests, whether, because it’s the receptionist telling me, I don’t – I do have a bit of concern whether she’s misread them or not read them properly. Whereas if it was someone who specialised, you know, in that kind of thing and they’re ringing you, then you know, I think you just have a bit more confidence.”* (Patient 5)

Some patients also mentioned the appeal of receiving results from someone who could immediately answer questions and arrange treatment. Patients expected specialist nurses to have more expertise than GPs, which was reassuring:

*“If it was with a specialist it would probably make you feel better. So, the doctor, when I was speaking to her, she didn’t really know a lot. So sometimes, if they don’t know a lot and they’re not very certain themselves, it makes you uncertain. Whereas if you get a phone call from someone that knows what they’re talking about, it makes you a little bit more at ease.”* (Patient 4)

Patients were also aware of GP workloads, and expected a specialist nurse to have more time to discuss results:

*“I’d prefer to speak to someone whose specific role was to speak to people about this, rather than a GP who has got many other responsibilities and might not give you the right time to discuss it.”* (Patient 11)

HCPs referred to the fact that the NLTM service was already in place and working well for younger patients via the NCSP and saw extending this to everyone as positive, being able to provide timelier follow-up, and better partner notification.

“*But it’s quite nice to know that if it was positive then there’s another service going into the notification and making sure they get their treatment and stuff. Because … sometimes it’s quite hard to get hold of a patient and, you know, I’m only in here two days a week.”* (GP 15)

#### Views on partner notification

HCPs expressed the view that partner notification in primary care could be “*a bit haphazard*” (GP 2), and that NLTM would be better placed to manage this.

*“*[GP’s name] *who was very good … but he said actually, more often than not, he would refer* [partner notification]*. But it’s simply because of time management that he just didn’t have time to sort it all out, and we didn’t have anybody in admin or any nurse, you know, that would sort of – he could palm that off.”* (Nurse 13)

While no patients objected to being asked for details of partners, views on the best way to manage partner notification varied. While some wished to notify partners themselves, others who expressed a view regarding whether this discussion was with their GP or the NLTM were either neutral, or preferred NLTM:

Patient 8: *I’d be quite happy, if I knew I’d got some infection or something I’d be quite happy to say, ‘Well it’s this person, that person or that person,’ you know.*

Interviewer: *And would you find it easier to be talking to a specialist nurse about that or to be talking to your GP?*

Patient 8: *Specialist nurse definitely, yeah.*

## Discussion

The majority of eligible patients in intervention practices were managed via NLTM. This demonstrates the feasibility of implementing an alternative clinical pathway allowing primary healthcare to elect for patients testing for chlamydia and gonorrhoea to be managed remotely by specialist nurses. NLTM was acceptable to both HCP and patients. Patients perceived benefits of NLTM to be a faster and a more proactive approach to communicating test results. The convenience and greater anonymity of a telephone consultation and being managed by a sexual health specialist was welcomed. HCPs expected the impact of NLTM on workload in primary care to be positive and to provide benefits for patients in relation to better and timely follow-up - particularly with regards to partner notification, which is essential for comprehensive case management [10, 11, 28]. We identified a need for improved clarity of NLTM pathway process for both patients and HCPs, and HCPs also expressed a desire for timely notification of actions taken by the NLTM. Without such feedback, HCPs often felt obligated to follow up positive test results themselves.

### Strengths and limitations

Qualitative interviews, with constant communication within the study team, helped to identify issues early and iteratively inform intervention implementation. Although 40% of eligible patients did not provide consent for NLTM, qualitative findings suggest time to conduct consent and frustration with consent procedures, rather than patients refusing consent, was the main reason for this. Our data suggests that Chlamydia test positivity was higher amongst patients excluded from the study. However, the study lacked power to estimate such a difference and there was considerable overlap of confidence intervals of the relevant prevalence estimates. A minority of patients were assigned to the incorrect study arm through the ICE interface when requesting chlamydia and gonorrhoea tests through the ICE online system. The initial cumbersomeness of the ICE system also caused frustration for HCP when conducting online consent. Interview findings demonstrates that in some cases patient consent was not obtained due to time pressures of practitioners rather than participant choice, particularly in intervention practices.

We recorded a chlamydia positivity rate of 2.6%, which is lower than that observed in the NCSP and probably reflects that the population tested had a mean age of 33 years, as chlamydia prevalence declines with age over 25 years [29]. Primary care can play an important role in opportunistic testing of at-risk individuals for chlamydia and gonorrhoea. Patients in our study valued consulting primary care for STIs in line with previous research [30], as GUM clinics may remain a stigmatised service for some [31]. However, our findings suggest opportunities may continue to be missed to diagnoses STIs in primary care, with research suggesting up to 40% of those attending GUM clinics, initially presenting at their general practice [32, 33]. Patients valued being contacted by a sexual health specialist via the NLTM over a GP, who may have less knowledge of STIs. Patient perceptions may be well founded, as a recent survey revealed that while 88% of GPs were confident in treating chlamydia, only 27% were confident treating gonorrhoea [34].

### Implications for research and/or practice

Our study suggests that the NLTM has the potential to provide a better service for patients whilst decreasing primary care workload and contribute to better use of clinical resources and better patient outcomes. Of particular benefit will be the improvement of partner notification and treatment of infected partner(s), and repeat testing 3 months after treatment for persons under 25 years old with chlamydia which can be facilitated through the NLTM and which may otherwise be overlooked.

Whilst an RCT is ideal to test the effectiveness of NLTM versus usual care, as NLTM approaches are already being implemented in this fast-moving area, real world evaluation based on mixed methods including routine data is needed.

Several improvements to the implementation process, to support HCPs and safeguard patients, were identified. HCPs often did not find it feasible to use the online ICE system during the consultation, thus the interface design, and training, should take this into account. The process of result notifications from the laboratory to the NLTM should be automated, and a feedback mechanism put in place so HCPs can check when their patients have been contacted about results and appropriately managed.

## Conclusions

There is sufficient evidence to support the wider implementation of this NLTM approach to managing common STI diagnosed in primary care. The fact that such implementation is already taking place across England suggests that large scale experimental evaluation of the pathway may now be infeasible. This notwithstanding, “real world” evaluation of the cost-effectiveness of the remote, nurse-led pathway compared to practice-based management is still needed and could be undertaken at relatively low cost using routine data.

## Data Availability

Data collected and analysed during the current study may be available from the corresponding author on reasonable request.

## Abbreviations

GUM: Genitourinary medicine
HCPs: Health care providers
ICE: Integrated Clinical Environment
NCSP: National Chlamydia screening programme
NLTM: Nurse-led, telephone management
STIs: Sexually transmitted infections
